# Computer-aided diagnosis of primary membranous nephropathy using expert system

**DOI:** 10.1186/s12938-023-01063-5

**Published:** 2023-02-02

**Authors:** Jie Gao, Siyang Wang, Liang Xu, Jinyan Wang, Jiao Guo, Haiping Wang, Jing Sun

**Affiliations:** 1 Department of Nephrology, Shandong Provincial Hospital Affiliated to Shandong First Medical University, Jinan, China; 2 953th Hospital, Shigatse Branch, Army Medical University (Third Military Medical University), Shigatse, China; 3 Department of Scientific Research, Shandong Provincial Hospital Affiliated to Shandong First Medical University, Jinan, China

**Keywords:** Expert system, Belief rule-based system, Primary membranous nephropathy, Membranous nephropathy

## Abstract

**Background:**

The diagnosis of primary membranous nephropathy (PMN) often depends on invasive renal biopsy, and the diagnosis based on clinical manifestations and target antigens may not be completely reliable as it could be affected by uncertain factors. Moreover, different experts could even have different diagnosis results due to their different experiences, which could further impact the reliability of the diagnosis. Therefore, how to properly integrate the knowledge of different experts to provide more reliable and comprehensive PMN diagnosis has become an urgent issue.

**Methods:**

This paper develops a belief rule-based system for PMN diagnosis. The belief rule base is constructed based on the knowledge of the experts, with 9 biochemical indicators selected as the input variables. The belief rule-based system is developed of three layers: (1) input layer; (2) belief rule base layer; and (3) output layer, where 9 biochemical indicators are selected as the input variables and the diagnosis result is provided as the conclusion. The belief rule base layer is constructed based on the knowledge of the experts. The final validation was held with gold pattern clinical cases, i.e., with known and clinically confirmed diagnoses.

**Results:**

134 patients are used in this study, and the proposed method is defined by its sensitivity, specificity, accuracy and area under curve (AUC), which are 98.0%, 96.9%, 97.8% and 0.93, respectively. The results of this study present a novel and effective way for PMN diagnosis without the requirement of renal biopsy.

**Conclusions:**

Through analysis of the diagnosis results and comparisons with other methods, it can be concluded that the developed system could help diagnose PMN based on biochemical indicators with relatively high accuracy.

**Graphical Abstract:**

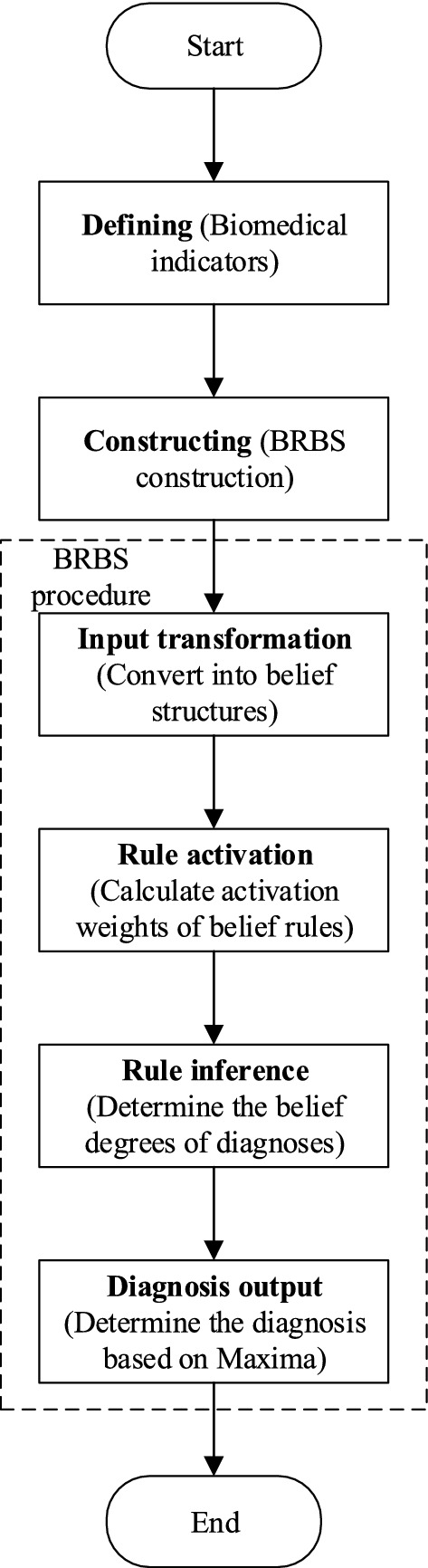

## Introduction

Membranous nephropathy (MN) is an organ-specific autoimmune disease and a leading cause of nephrotic syndrome (NS) in adults [1]. Approximately 75–80% of MN consists of cases with unknown etiology [2], namely, primary MN (PMN) and idiopathic MN (IMN), and the remaining incidences caused by other conditions, known as secondary MN (SMN), which includes cancers, infections such as hepatitis B, autoimmune diseases such as systemic erythematosus, or drugs.

The clinical manifestations of MN are extremely heterogeneous. Patients with PMN can present with various degrees of proteinuria. Clinically, 80% of patients present with nephrotic syndrome with the typical clinical features including nephrotic-range proteinuria (>3.5 g/day), hypoalbuminemia, hyperlipidemia, and edema [3–5]. In addition, it has been found in a previous study that in 116 patients, 55% of patients have microscopic haematuria [6]. Furthermore, the disease also exhibits heterogeneous outcomes. Approximately 30% of cases can be relieved spontaneously, while 30–40% of patients develop end-stage kidney disease within 5-15 years of onset [7]. Most importantly, mortality from MN is high because of complications such as infections, cardiovascular events, or malignancies [8]. In view of the complexity of clinical manifestations and prognosis of PMN, how to make an accurate diagnosis is the primary factor to control the progress of the disease.

Renal biopsy is considered to be the gold standard in the diagnosis of MN, however, the procedure still carries a low but not negligible mortality rate, is associated with a significant risk of hemorrhage, and may cause considerable discomfort [4, 9, 10]. Recently, with the identification of podocyte antigens and associated autoantibodies, MN can now be classified according to antigenic specificity, and most PMN is mediated by antibodies to the M-type phospholipase A2 receptor (anti-PLA2R) (85%), thrombospondin type 1 domain containing 7A (THSD7A) (3–5%), or by other as yet unidentified mechanisms (10%), which could be analyzed based on biochemical indicators [11, 12]. Therefore, it is possible to make a diagnosis of PMN based on the biochemical indicators of patients without the requirement for an invasive renal biopsy, and the possibility of replacing the invasive renal biopsy for the diagnosis of PMN is becoming an increasingly realistic option.

However, though diagnosing PMN based on certain biochemical indicators has shown to be effective, it is not without challenges. Firstly, as patients with PMN are not necessarily to have the same or even close biochemical indicators, it often relies heavily on the clinicians to make a diagnosis based on their understanding of these biochemical indicators. Indeed, as different clinicians would often have different backgrounds and experiences with PMN, the diagnosis provided by them on the same patient may not always be the same, thus, how to effectively establish a mechanism that could effectively capture the experiences and knowledge of the experts on the diagnosis of PMN remains an urgent yet realistic issue. Secondly, it is often inevitable that some patients tend not to conduct complete examinations of all relevant biochemical indicators, either because of practical reasons or economic reasons, and the values of some biochemical indicators could be missing, which could significantly increase the difficulty to provide a reliable diagnosis. Therefore, how to provide a relatively reliable diagnosis of PMN with incomplete information is another challenge that needs to be addressed.

Many artificial intelligence methods have been applied to medical diagnosis problems, including conventional neural networks, fuzzy sets and expert systems. For instance, Ref. [13] treated medical diagnosis problems as pattern recognition problems, and adopted hesitant fuzzy sets for medical diagnosis. Ref. [14] proposed a novel medical diagnosis method by combining fuzzy rule-based approach, harmony search algorithm, and heuristic algorithm. Ref. [15] proposed tuned fuzzy kNN based on uncertainty classifiers (TFKNN) for diabetes diagnosis to increase diagnosis precision. Among these methods, expert systems have shown to be effective and reliable in modeling the knowledge of experts on different aspects and emulating the decision-making process of humans, and have been widely regarded as an effective tool to deal with medical diagnosis problems [16–21].

Among various expert systems, the belief rule-based system, which is a novel artificial intelligence method that combines fuzzy rule base and evidence theory, has received increasing attention for its advantages in modeling the knowledge of the experts under different kinds of uncertainty [22–24]. It consists of two parts, namely, a belief rule base (BRB) that models the knowledge and judgments of the experts, and the inference engine that produces an inferential result for any given inputs based on the belief rule base. Because of the existence of the belief rule base and inference engine in the belief rule-based system, unlike most artificial intelligence methods, its reasoning process and inferential results are traceable and explainable, more suitable for medical diagnosis problems [25–29]. Moreover, as the belief rule base can be constructed with partial information, the belief rule-based system is well-suited for reasoning with missing information, which makes it suitable for this problem.

Motivated by the above challenges, a belief rule-based expert system is established for PMN diagnosis in this paper, where 9 different biochemical indicators, including albumin, total cholesterol and triglycerides, are used as the input variables of the belief rule base, and the belief rule base is then constructed based on the knowledge of experts. For any patient to be diagnosed, the inference engine could produce the inferential result and diagnosis conclusion based on the biochemical indicators of the patients. Results show that the proposed method could provide reliable and accurate diagnosis results, and could achieve better performance compared with other methods.

## Results

The diagnosis system was implemented with data from Shandong Provincial Hospital Affiliated to Shandong First Medical University. It is noteworthy that at the end of the diagnosis, the system not only presents the diagnosis result, which indicates the PMN condition of the patient, but it also shows the number of activated belief rules to this specific situation, thus could support the expert to understand the conclusion provided by the system by tracing back the activated belief rules and the activation weights. The use of belief rule-based system allows the traceability of the diagnosis results through the inference process.

### Basic characteristics

The patient group used in this study consists of 134 patients, including 32 (23.88%) patients without PMN, 61 (44.52%) patients with stage I PMN, and 41 patients (31.60%) with Stage II PMN. The mean age for patients without PMN is 37.2 ± 15.1 years, while the mean age for patients with stage I PMN is 45.5 ± 20.4 years and the mean age for patients with stage II PMN is 43.2 ± 18.8 years. For patients without PMN, 46.9% are male, while 52.5% are male for patients with stage I PMN, 48.8% are male for patients with stage II PMN. A detailed comparison of statistic data of the patients is listed in Table 1.

**Table 1:** Demographic characteristics

Parameter	Non-PMN (*n*=32)	Stage I PMN (*n*=61)	Stage II PMN (*n*=41)
Age (years)	37.2 ± 15.1	45.5 ± 20.4	43.2 ± 18.8
Gender (male, %)	15 (46.9%)	32 (52.5%)	20 (48.8%)

### Diagnosis performance of BRBS

The performance of BRBS is defined by sensitivity, specificity, and accuracy. For the entire patient group, the percentage of correctly classified patients, i.e., the accuracy is 97.8%, the percentage of true positives, i.e., the sensitivity is 98.0%, and the percentage of true negatives, i.e., the specificity is 96.9%. As for patients of each individual group, for patients without PMN, the accuracy is 96.9%, for patients with stage I PMN, the accuracy is 98.4%, and for patients with stage II PMN, the accuracy is 97.6%. The diagnosis accuracy of BRBS for male is 98.1% and for female is 97.6%, with no significant differences. The detailed performance of BRBS is listed in Table 2, and the quality of diagnosis according to different parameters is listed in Table 3.

**Table 2:** Diagnosis performance of different methods

	FRBS (%)	TFKNN (%)	BRBS (%)
Sensitivity	93.2	94.5	98.0
Specificity	94.1	95.0	96.9
Accuracy	90.3	94.0	97.8

**Table 3:** The quality of diagnosis according to different parameters

		Accuracy (%)
*All*		97.8
Non-PMN		96.9
Stage I PMN		98.1
Stage II PMN		97.6
*Gender*		
Male		98.5
Female		97.0
*Age*		
15–30		95.0
30–45		98.0
45–60		98.4

The confusion matrix of the proposed method is shown in Table 4. From Table 4, it can be found that among all 134 patients, 3 cases are misdiagnosed by the proposed method, which is relatively small compared to the total number of cases. It is also worth noting that all misdiagnosed PMN cases are misdiagnosed to non-PMN, which are relatively close. Hence, it can be said that the proposed method could provide reliable and reasonable diagnosis results for PMN.

**Table 4:** Confusion matrix of the proposed method

	Predicted		
	Non-PMN	Stage I PMN	Stage II PMN
*Actual*			
Non-PMN	31	0	1
Stage I PMN	1	60	0
Stage II PMN	1	0	40

**Fig. 1:**
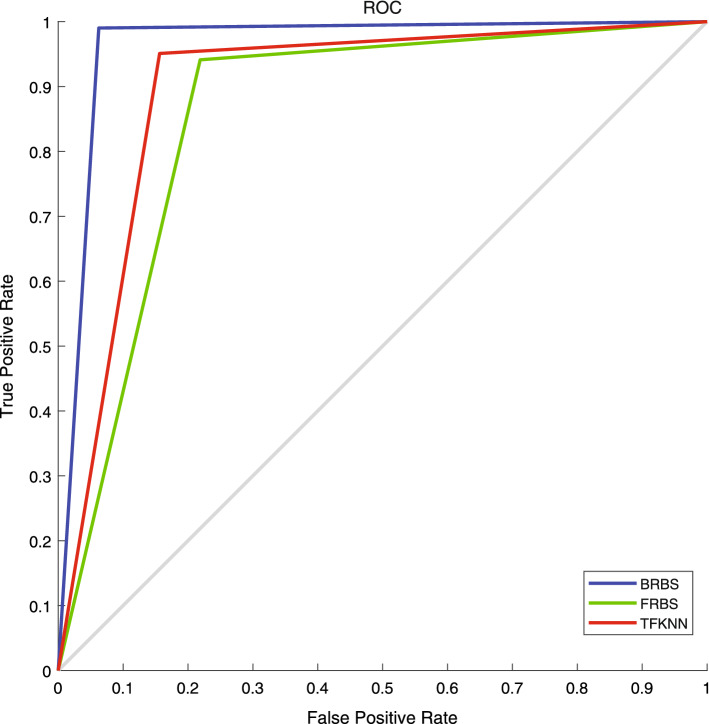
ROC curve of the diagnosis performance. Blue line is the ROC curve of the method in this study. Green line is the ROC curve of FRBS. Red line is the ROC curve of TFKNN

Receiver operating characteristic (ROC) curves are created for BRBS, FRBS and TFKNN, and the AUCs of different methods are measured to analyze the performance of BRBS, as shown in Fig. 1. From Fig. 1, it can be found that the AUC of BRBS is 0.9283, higher than those of other methods.

From the comparison analysis with FRBS and TFKNN, it can be found that the proposed method in this study has obviously better performance, either for accuracy or for AUC. Furthermore, compared with other methods, the proposed method could have better interpretability and traceability due to the application of belief rules. Therefore, from the comparison results, it can be said that the proposed method provides an effective, efficient and non-invasive way to diagnose PMN.

### Sensitivity analysis

Sensitivity analysis could provide the basic idea of how different input indicators affects the diagnosis results. For the entire group of patients, by removing the values of different indicators and conducting the diagnosis approach, the resulting diagnosis accuracy is listed in Table 5.

**Table 5:** Sensitivity analysis

Indicator	Accuracy (%)
ALB	94.0
TC	93.3
TG	94.0
BUN	94.8
CREA	97.0
eGFR	96.3
Urine SG	94.8
Urine RBC	94.0
Proteinuria	89.6

It should be noted that for the diagnosis system, missing certain indicator values would not significantly affect its performance, as the overall accuracy is still satisfactory for most cases, higher than that of FRBS. For some special cases where certain biochemical indicators may not be available, the diagnosis system could provide relatively reliable diagnosis results.

## Discussion

Primary membranous nephropathy (PMN) constitutes a large part of membranous nephropathy, which could lead to nephrotic syndrome (NS) in adults. The diagnosis of PMN based on the biochemical indicators of patients without the requirement for an invasive renal biopsy has attracted extensive interest in recent years, and how to develop a diagnosis method for PMN with biochemical indicators has become an important issue. In this study, we present the BRBS that automatically diagnoses patients suspected of PMN to different stages based on biochemical indicators. Nine typical indicators, including albumin (ALB), total cholesterol (TC), triglycerides (TG), blood urea nitrogen (BUN), creatinine (CREA), estimated glomerular filtration rate (eGFR), urine specific gravity (urine SG), urine red blood cells (urine RBC), and proteinuria are used, and the proposed method achieved an overall accuracy of 97.8%, sensitivity of 98.0% and specificity of 96.9% for diagnosis of PMNs from biochemical indicators. Our method provides a feasible, effective and efficient way for the diagnosis of PMN without the invasive biopsy at an early stage, which could help patients receive necessary treatment while reducing harm.

PMN can be characterized as an organ-specific autoimmune disease with unknown etiology, which could potentially lead to nephrotic syndrome. Due to the heterogeneous clinical manifestations of PMN, its diagnosis has been challenging, and it can be misdiagnosed as IMN or other nephropathy diseases. Moreover, the progress of PMN could go on for years, and early detection of PMN has been difficult. On the other hand, the gold standard for the diagnosis of PMN, i.e., renal biopsy, could cause considerable discomfort and may even lead to a low yet nonnegligible mortality rate. Developing a low-cost, non-invasive method for the diagnosis of PMN has become an important issue.

With recent development in the identification of podocyte antigens and associated autoantibodies of PMN, it has become possible to provide relatively reliable diagnosis of PMN based on several biochemical indicators, and that coincidences with the rapid development of artificial intelligence (AI) technology. By adopting proper AI techniques, it is not only possible, but also effective to provide accurate diagnosis results for patients with PMN. Moreover, an expert system-enabled AI technique could sufficiently support the interpretable diagnosis of PMN disease with the support of clinician knowledge, and the BRBS-based diagnosis technique in our study achieves an AUC of 0.93, sensitivity of 98.0%, specificity of 96.9%, and overall accuracy of 97.8%. [30] adopted the fuzzy rule-based system for medical diagnosis by constructing fuzzy rule base from the previous knowledge. By testing with FRBS, the AUC of FRBS is 0.74, and the sensitivity, specificity and overall accuracy are 93.2%, 94.1% and 90.3%, respectively. In this study, we used belief rule to represent the clinician knowledge, which is different from the fuzzy rule. Firstly, the belief rule used in this study is capable of modeling the preference knowledge of clinicians with uncertainty and hesitancy, which is a common problem in the modeling and representation of prior knowledge. Secondly, this study selects indicators that are highly relevant to the diagnosis of PMN, and the diagnosis results are presented in terms of disease stages, which could provide more information for both the clinicians and the patients. Moreover, the proposed method also outperforms TFKNN, where the the sensitivity, specificity and overall accuracy of TFKNN are 94.5%, 95.0% and 94.0$, respectively. However, it is worth noting that all these methods could benefit clinicians in diagnosing PMN using biochemical indicators in a timely and feasible manner, and could avoid invasive renal biopsy, which could ease the pain of patients and reduce the expanses.

One significant advantage of the proposed method is that through the construction of the BRB, the diagnosis process becomes a white-box process with high interpretability and traceability. Unlike other machine-learning methods, the diagnosis based on the belief rule-based system can be viewed as the reference among historical cases and knowledge of experts, which not only enables the diagnosis results to be theatrical sound, but also ensures the reliability of the diagnosis results. Thus, when the proposed method produces a diagnosis result, the clinicians could trace back the diagnosis to certain rules, understanding the reason for this diagnosis. Compared with other methods, it can be said that the proposed method is more suitable for diagnosis problems.

On the other hand, due to the application of the disjunctive BRBS, the proposed method is capable of dealing with incomplete information, that is, when a patient does not have the testing results for all biochemical indicators, a diagnosis result could still be reached. As some patients may fail to conduct all examinations prior to the medical treatment, the proposed method could provide preliminary diagnosis results as references for the medical practitioners, thus reducing their burden.

It is worth noting that the proposed method is effective in diagnosing most PMN cases, not only for diagnosing whether a patient has PMN, but also for determining the exact stage of PMN. As shown in the results, among 134 tested cases, 131 were correctly diagnosed to the exact stage, and all the misdiagnosed patients with PMN were misdiagnosed to non-PMN instead of different stages, which further shows the effectiveness and reliability of the proposed method. However, it is also worth noting that as stage III and IV PMN patients are not included in the case study, the proposed method is not validated for these PMN stages. More studies with stage III and IV PMN patients could help validate and improve the proposed method.

Moreover, adapting a novel method for medical applications is always challenging, and there are some limitations to our method. Firstly, the proposed method is mainly used as a preliminary diagnosis practice, when patient is diagnosed as PMN with high probability, renal biopsy may be needed some time. Secondly, the performance of the proposed method could be further improved if more data are available, and that could be difficult due to the privacy protection of patient data.

## Conclusion

Focusing on the problem of primary membranous nephropathy diagnosis, a belief rule-based expert system is introduced in this paper in order to deal with uncertainty and missing information in PMN diagnosis. As the diagnosis of PMN relies heavily on the knowledge of the experts, the belief rule base is constructed based on the knowledge of the experts, where a set of 9 biochemical indicators are selected as the input variables and the diagnosis result is used as the output. The inference engine of the conventional belief rule-based system is modified to suit the PMN diagnosis problem, and the diagnosis could be obtained based on the biochemical indicators of the patient using the belief rule-based expert system. Results with real-world patients show that the proposed method could reach 97.8% accuracy, significantly higher than other methods. Therefore, this study presents a reliable and effective decision-support platform to clinicians for the diagnosis of primary membranous nephropathy. For future studies, we will further investigate the possibility of training the parameters of the belief rule base to improve its performance.

## Methods

The object of this work is to develop a medical decision support system using belief rule-based system for the diagnosis of PMNs based on biochemical indicators. The effects of this system could be significant, avoiding invasive renal biopsy and allowing the use of expert knowledge in the diagnosis process.

Data used in this study were collected in 2021 under the approval of the Ethics Committee of the Shandong Provincial Hospital Affiliated to Shandong First Medical University. A total of 134 patients are included in this study. 102 presented PMN, and 32 are healthy, composing the control group. The patients with PMN are divided into two groups: (1) patients with stage I PMN, and (2) patients with stage II PMN.

Renal biopsy and exams were conducted at Shandong Provincial Hospital Affiliated to Shandong First Medical University, and the following indicators are included: albumin (ALB), total cholesterol (TC), triglycerides (TG), blood urea nitrogen (BUN), creatinine (CREA), estimated glomerular filtration rate (eGFR), urine specific gravity (urine SG), urine red blood cells (urine RBC), and proteinuria. All patients were given written consent, and this study is in agreement with The Declaration of Helsinki.

### Data processing

By performing examinations on the patients, the following indicators are included: albumin (ALB), total cholesterol (TC), triglycerides (TG), blood urea nitrogen (BUN), creatinine (CREA), estimated glomerular filtration rate (eGFR), urine specific gravity (urine SG), urine red blood cells (urine RBC), and proteinuria. In addition, renal biopsy is performed on the patients, based on the results of renal biopsy, patients are divided into two groups: patients diagnosed with PMN and patients without any signs of PMN. For patients in the PMN group, the inclusion criteria are as follows: (1) patients are included if they are diagnosed with PMN clinically, (2) patients are diagnosed with PMN by examinations. For the control group, patients who are evaluated without evidence of PMN based on examinations and medical records are included. The characteristics of the patient data are summarized in Table 6.

**Table 6:** Data statistics

Status		Value								
		ALB	TC	TG	BUN	CREA	eGFR	Urine SG	Urine RBC (HPF)	Proteinuria
*Non-PMN*										
Maximum		48.100	7.160	8.280	10.800	92.300	132.400	1.035	5.600	1.000
Minimum		30.900	2.750	0.440	2.900	43.100	86.500	1.004	0.100	0.000
Mean		43.772	4.362	1.561	5.431	68.847	118.270	0.955	1.156	0.172
*PMN*										
Maximum		44.700	19.310	19.810	11.300	100.000	152.000	1.055	101.700	4.000
Minimum		12.400	4.270	0.510	2.200	30.000	78.000	1.002	0.300	0.500
Mean		26.056	8.514	2.768	4.817	65.510	108.873	1.019	13.496	2.490

Based on the appearance of electron-dense deposits in glomerular basement membrane (GBM) in electron microscopy, PMN can be classified into 4 stages. During the initial stages, podocyte effacement is noted with minimal to no changes in the GBM (stage I). If the deposits persist, new basement membrane material is laid between these immune deposits giving rise to the spike formations identified on methenamine silver stains which are readily observed on electron microscopy (stage II). In stage III, these deposits are completely encircled by newly laid basement membrane. In more advanced stages, basement membranes are thickened, and the deposits become more lucent and the spikes become less apparent. Our patients included only stage I and stage II, According to the renal biopsy results, among all patients with PMN, 61 patients were diagnosed with stage I and 41 with stage II, and no patients with pathological stage III–IV. Moreover, there are several non-PMN patients included in this study to further validate our approach. The distribution of patients are listed in Table 7.

**Table 7:** Distribution of patients

	Number of patients
Non-PMN	32
Stage I PMN	61
Stage II PMN	41

### Belief rule-based system

The diagnosis of PMN based on the indicators can be defined as a typical classification problem as there are finite, real-valued indicators and a classification label. A patient may either have PMN, either Stage I, labeled as “1”, or Stage II, labeled as “2”, or Stage III, labeled as “3”, or Stage IV, labeled as “4”, or the patient may have no sign for PMN and labeled “0”. Based on the annotated patient data, the relationship among the feature values, i.e., the examination results, and the actual diagnosis could be determined. Moreover, as the diagnosis process is inherently an expert-based process, the knowledge of the experts should also be taken into consideration to help increase the accuracy and reliability of the diagnosis. The belief rule-based system is a novel artificial intelligence method that could model the cognitive process of humans, and has been adopted to various classification problems. It is an expert system made up of numerous belief rules that each represent prior information on the relationship between the input and the consequent. The belief rule-based system is implemented by using MATLAB 2019b, and all the experiments are conducted on Core(TM) i5-7400 CPU @ 3.00 GHz with Windows 10.

**Fig. 2:**
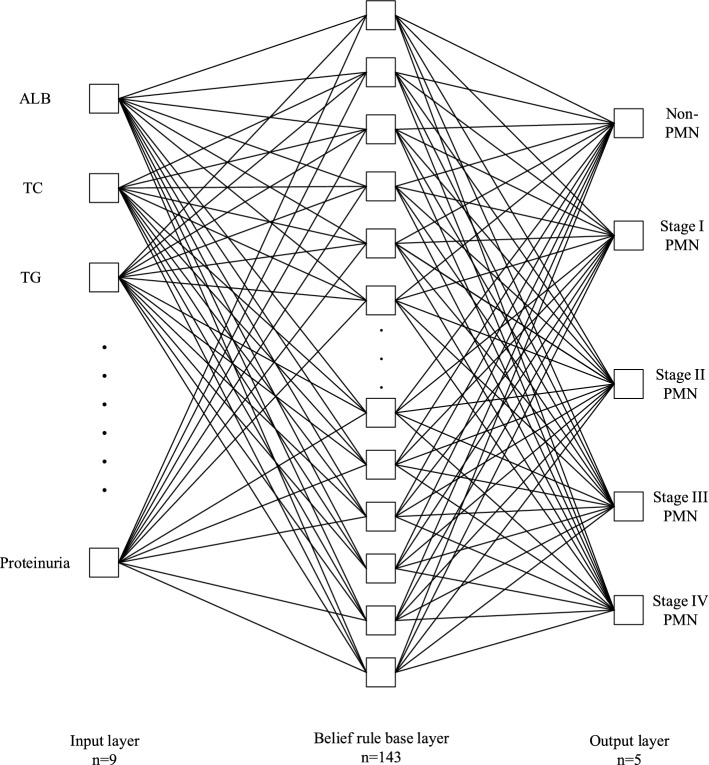
Belief rule-based system structure. Periodontal chart with input layer (*n* = 9), belief rule base layer (*n* = 143) and output layer (*n* = 5) which refers to periodontitis grading. Albumin, total cholesterol, triglycerides, blood urea nitrogen, creatinine, estimated glomerular filtration rate, urine specific gravity, urine red blood cells, and proteinuria are taken into account. For each patient, a set of 9 inputs is produced, and the output layer consists of four stages (I, II, III, and IV) and non-PMN will produce the diagnosis result

In medical diagnosis, it is of huge significance to process inaccurate information and provide traceable and interpretable diagnosis results. One possible and effective approach for this requirement is the application of belief rule-based system to model and process the uncertain experts knowledge. In previous studies, it has been found that the application of belief rule-based system could have many benefits, including the acquisition of experts knowledge, the construction of belief rule base and the automated process of the diagnosis, and could achieve relatively promising results. The core idea of the belief rule-based system is the capture of experts’ knowledge in the form of belief rule base, thus assisting the diagnosis process with the help of experts’ knowledge. With the application of belief rule-based system, we developed a medical decision support system for diagnosing PMN, shown in Fig. 2.

The belief rule-based medical diagnosis system consists of three layers, input layer, belief rule base layer, and output layer. For the input layer, there are nine nodes, and the input for the belief rule-based system could be transformed into corresponding belief distributions in this layer. The belief rule base layer, which consists of numerous belief rules that represent the relationship between the indicators and the classification, is the main component of the belief rule-based system, as it stores the prior knowledge for the diagnosis, and there are 143 nodes in the belief rule base layer, each corresponding to one belief rule. The output layer is consisted of five nodes, corresponding to different diagnoses, i.e., non-PMN, stage I, stage II, stage III and stage IV.

#### Input layer

**Fig. 3:**
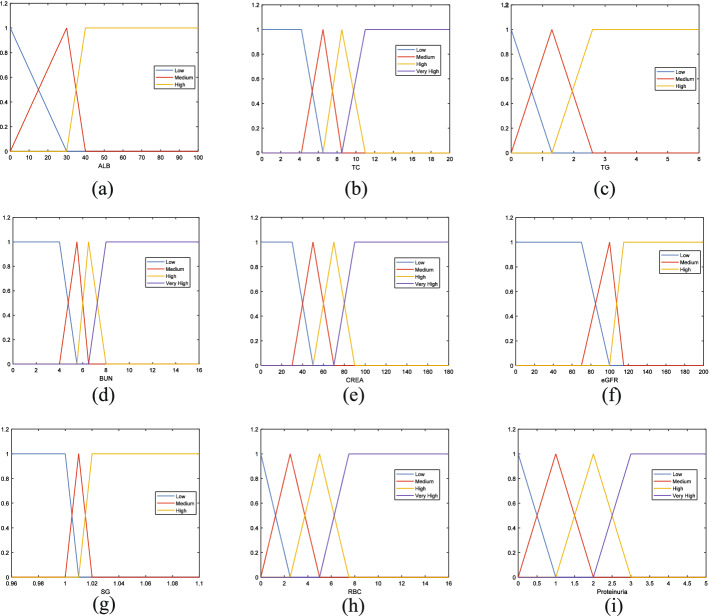
Transformation functions for biochemical indicators used in this study. **a** Transformation function for albumin. **b** Transformation function for total cholesterol. **c** Transformation function for triglycerides. **d** Transformation function for blood urea nitrogen. **e** Transformation function for creatinine. **f** Transformation function for estimated glomerular filtration rate. **g** Transformation function for urine specific gravity. **h** Transformation function for urine red blood cells. **i** Transformation function for proteinuria

The belief rule-based systems use information from patients’ examinations as the input, including albumin (ALB), total cholesterol (TC), triglycerides (TG), blood urea nitrogen (BUN), creatinine (CREA), estimated glomerular filtration rate (eGFR), urine specific gravity (urine SG), red blood cells (urine RBC), and proteinuria. The numerical values of these indicators are transferred to belief distributions following the distribution of the data of each indicator, and there are nine different transformation functions corresponding to nine indicators. Thus, for each patient, its examination results could be transformed into corresponding belief distributions to be used for diagnosis. The transformation functions of different indicators are illustrated in Fig 3.

For instance, ALB has three grades, described by the reference grades Low, Medium and High, respectively, expressed as:

Low ALB ($$ALB_{/text{Low}}$$) < 30 $$\rightarrow $$ $$ALB_{\text{Low}}=\{(0,1),(0,1),(30,0)\}$$.

Medium ALB ($$ALB_{/text{Medium}}$$) 30-40 $$\rightarrow $$ $$ALB_{/text{Medium}}=\{(0,0),(30,1),(40,0)\}$$.

High ALB ($$ALB_{\text{High}}$$) > 40 $$\rightarrow $$ $$ALB_{\text{High}}=\{(30,0),(40,1),(70,1)\}$$.

Similarly, the transformation functions are built for all indicators, and all relevant function data, i.e., values that determine the belief degree for the functions, are determined by the experts. It is also worth noting that in this study, the most widely used triangular and trapezoidal transformation functions are used as they could better represent the situation.

#### Belief rule base layer

**Fig. 4:**
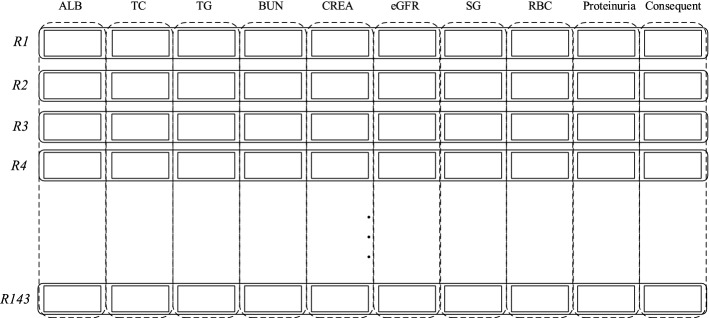
Belief rule base layer. Albumin, total cholesterol, triglycerides, blood urea nitrogen, creatinine, estimated glomerular filtration rate, urine specific gravity, urine red blood cells, and proteinuria are used as the input. The consequent represents the probability of different outputs, i.e., non-PMN, stage I PMN, stage II PMN, stage III PMN, and stage IV PMN

The belief rule base is the core component of the belief rule-based system, where numerous belief rules are constructed to model the human cognition process. The belief rules in the belief rule base are assembled as “IF $<conditions>$, Then $<conclusion>$”. In this study, the belief rule base is set up based on the knowledge of experts using nine attributes ($<conditions>$) and one consequent ($<conclusion>$), where each attribute corresponds to one indicator and the consequent corresponds to the diagnosis result. Each belief rule represents a piece of knowledge with regard to the diagnosis of PMN, either from historical data or from the knowledge of experts, as illustrated in Fig 4.

For instance, one belief rule is expressed by:

# Rule 1: If high ALB or low TC or low TG or low BUN or moderate CREA or high eGFR or low urine SG or low urine RBC or low proteinuria, then there is $90\%$ confidence non-PMN and $10\%$ stage I PMN.

One important note is that the belief rules in constructed belief rule base shall cover all possible combinations of this problem to assure its effectiveness and the consistency of the rules will be reviewed to avoid inconsistent rules. The belief rule base is constructed through extensive meetings and discussion, and with the support of historical data.

For each patient, once its examination results are transformed in the input layer, the belief rule base layer will work as a reference, as one or more belief rules that are related to the input are activated by the input information, i.e., the diagnosis of the patient would be determined on the basis of these belief rules. The degree to which each belief rule is activated is determined using the weight activation function, which is determined by the closeness between the input and the rule and the weight of the belief rule. Through rule activation mechanism, and input is matched by its related belief rules in the belief rule base layer, thus could be used as the basis for determining the diagnosis result in the output layer.

In this study, the disjunctive belief rule is used, and the activation weight of each belief rule is calculated based on the sum of the individual matching degree of different indicators as [31]:

$$\begin{aligned} \begin{aligned} \omega _k=\frac{\theta _k\sum _{m=1}^M{(\alpha _m^k)}}{\sum _{l=1}^L{\theta _l \sum _{m=1}^M{(\alpha _m^l)}}}, \end{aligned} \end{aligned}$$

where $\omega _k$ represents the activation weight of the *k*th belief rule, $\alpha _m^l$ represents the individual matching degree of the *m*th indicator of the *k*th belief rule, and $\theta _k$ represents the weight of the *k*th belief rule.

#### Output layer

The output layer uses activated belief rules from the belief rule base layer to determine the diagnosis result, namely, either non-PMN, stage I, stage II, stage III, or stage IV. The output layer is activated by using evidential reasoning algorithm, where the consequents of all activated belief rules and the activation weights are considered, and the probability of different diagnosis results $\beta _n$ could be provided as:

1 $$\begin{aligned} \begin{aligned} \beta _n=\frac{d\left[ \prod _{k=1}^L{\left( w_k\beta _{n,k}+1-w_k\sum _{i=1}^N{\beta _{n,k}}\right) }-\prod _{k=1}^L{\left( 1-w_k\sum _{n=1}^N{\beta _{n,k}}\right) }\right] }{1-d\left[ \prod _{k=1}^L{\left( 1-w_k\right) }\right] } \end{aligned} \end{aligned}$$

with,

2 $$\begin{aligned} \begin{aligned} d=\left[ \sum _{n=1}^N{\prod _{k=1}^L{\left( w_k\beta _{n,k}+1-w_k\sum _{j=1}^N{\beta _{j,k}}\right) }}-(N-1)\prod _{k=1}^L{\left( 1-w_k\sum _{j=1}^N{\beta _{j,k}}\right) }\right] ^{-1}. \end{aligned} \end{aligned}$$

As different diagnosis results could be assigned to probability with different values, the diagnosis is determined using the Maxima technique, which is defined as the referential grade with the maximal belief degrees:

3 $$\begin{aligned} \begin{aligned} Con=D_n,\ n=\arg \max _n(\beta _n). \end{aligned} \end{aligned}$$

### Outcome of interest

To measure the performance of the diagnosis system, the accuracy of the diagnosis results is analyzed as the main indicator, which is measured by the percentages of correctly diagnosed patients among the entire group. In addition, sensitivity and specificity are also analyzed to more comprehensively describe the diagnosis performance, where sensitivity is measured by the proportions of true positives in patients with PMN, and specificity is measured by the proportions of true negatives in patients without PMN. Moreover, the receiver operating characteristic (ROC) curve is constructed, and the corresponding area under curve (AUC) is analyzed, where ROC curve is defined by the points of true positive rate, i.e., sensitivity, and false positive rate, i.e., 1 minus specificity at different threshold settings, and AUC describes the possibility of classifying a positive data with higher confidence than a negative data.

### Comparison analysis

Descriptive statistics are applied to analyze the clinical characteristics of patients used in this study, where the indicators are expressed by mean value and standard deviation. In order to verify the effectiveness and feasibility of the proposed method, the results of this study are compared with those using fuzzy rule-based system (FRBS) and tuned fuzzy KNN based on uncertainty classifiers (TFKNN).

For FRBS, the fuzzy rules are constructed based on the belief rules, where the consequents are changed to specific classifications instead of belief structures, and 143 fuzzy rules are constructed. For the input, relevant fuzzy rules are searched and fired according to their closeness to the input according to the rule firing scheme from previous studies. The results are obtained using Knowledge Extraction based on Evolutionary Learning (KEEL) [32] on Core(TM) i5-7400 CPU @ 3.00 GHz with Windows 10.

For TFKNN, the Euclidean distance is used to determine the distances between neighbors, and Ball tree is adopted to search for the nearest neighbors. The constructed 143 belief rules are converted and used as the training group to train the method, and the 134 patients are used as testing group to show its performance. The results are obtained by using Matlab 2019b on Core(TM) i5-7400 CPU @ 3.00 GHz with Windows 10.

## Data Availability

The data used in this study are available from the corresponding author upon reasonable request.

## Appendix

See Table 8.

**Table 8:** Belief rule base used in this study

No.	Antecedent attribute									Consequent				
	ALB	TC	TG	BUN	CREA	eGFR	Urine SG	Urine RBC	Proteinuria	Non-PMN	Stage I	Stage II	Stage III	Stage IV
1	H	L	L	L	M	H	L	L	L	90%	10%	0	0	0
2	M	M	H	L	H	L	M	H	VH	60%	30%	10%	0	0
3	M	H	M	VH	H	L	M	VH	H	30%	60%	10%	0	0
4	H	L	M	VH	H	L	L	M	M	10%	70%	20%	0	0
5	L	H	H	H	M	M	H	VH	H	0	80%	20%	0	0
6	L	M	L	L	M	L	M	M	L	80%	20%	0	0	0
7	M	L	M	H	VH	H	L	L	L	90%	0	10%	0	0
8	H	L	L	M	M	H	L	L	L	100%	0	0	0	0
9	H	H	M	VH	VH	H	H	VH	VH	0	0	0	20%	80%
10	L	H	M	H	VH	M	H	M	H	0	10%	85%	5%	0
11	L	L	M	L	M	L	L	L	M	70%	30%	0	0	0
12	M	M	H	M	H	L	H	M	L	20%	30%	50%	0	0
13	H	H	H	VH	H	M	H	L	H	0	0	20%	80%	0
14	M	VH	H	VH	H	M	H	VH	VH	0	0	10%	80%	10%
15	H	M	L	H	H	M	L	VH	H	0	0	0	20%	80%
16	M	VH	H	VH	L	H	M	VH	H	0	0	40%	60%	0
17	L	L	L	L	M	H	L	M	L	10%	90%	0	0	0
18	L	M	M	H	M	L	L	L	M	0	80%	20%	0	0
19	L	L	H	H	M	L	L	L	H	0	60%	40%	0	0
20	L	L	M	L	L	M	L	M	M	0	70%	30%	0	0
21	L	M	H	M	H	H	H	M	L	0	0	60%	40%	0
22	M	L	M	H	M	M	L	L	M	0	0	70%	30%	0
23	L	H	H	VH	M	H	L	L	H	0	0	0	70%	30%
24	L	M	H	H	M	H	M	L	H	0	0	70%	30%	0
25	M	L	L	M	M	L	L	M	L	10%	70%	20%	0	0
26	M	M	L	M	L	L	M	L	L	20%	80%	0	0	0
27	H	M	L	M	L	L	M	L	L	60%	40%	0	0	0
28	H	L	L	L	M	L	L	L	M	90%	10%	0	0	0
29	H	L	M	L	L	L	L	L	L	95%	5%	0	0	0
30	H	L	L	M	L	L	M	M	L	60%	30%	10%	0	0
31	H	M	L	M	L	L	L	L	L	80%	10%	10%	0	0
32	H	M	L	L	M	L	L	L	L	50%	30%	20%	0	0
33	H	L	M	L	L	H	H	VH	H	0	0	70%	30%	0
34	L	VH	H	VH	L	L	M	L	L	0	0	10%	70%	20%
35	L	H	H	VH	H	L	H	M	H	0	0	30%	70%	0
36	L	VH	M	VH	L	M	L	H	H	0	0	0	90%	10%
37	M	H	M	M	H	L	L	M	L	0	0	40%	60%	0
38	L	H	H	H	L	M	H	M	H	0	0	0	80%	20%
39	M	H	M	M	H	M	M	L	L	0	30%	70%	0	0
40	L	M	H	M	L	L	M	M	H	0	0	80%	20%	0
41	L	H	H	VH	H	M	H	VH	VH	0	0	0	40%	60%
42	L	M	H	M	H	H	H	VH	H	0	0	30%	70%	0
43	M	H	H	M	L	H	H	H	VH	0	0	60%	30%	10%
44	L	H	H	M	H	L	M	H	H	0	20%	80%	0	0
45	H	M	L	M	M	L	L	L	L	30%	60%	10%	0	0
46	H	H	M	L	M	L	H	H	L	0	50%	30%	10%	0
47	L	VH	H	VH	H	L	L	VH	VH	0	0	0	20%	80%
48	L	H	M	H	M	L	L	H	H	0	0	80%	20%	0
49	H	M	H	M	M	L	L	H	M	50%	40%	10%	0	0
50	L	M	L	M	L	L	H	M	H	0	70%	30%	0	0
51	M	H	L	H	M	H	H	H	H	0	30%	60%	10%	0
52	H	M	L	L	L	L	L	L	L	60%	40%	0	0	0
53	M	VH	H	VH	H	H	L	H	H	0	0	30%	50%	20%
54	L	H	M	H	H	L	H	VH	H	0	0	0	80%	20%
55	L	VH	H	L	L	H	L	VH	VH	0	0	70%	20%	10%
56	L	H	M	H	L	L	L	M	H	0	40%	60%	0	0
57	M	H	M	M	M	M	H	H	L	0	75%	25%	0	0
58	M	M	M	M	L	M	H	M	M	30%	70%	0	0	0
59	M	H	L	M	H	L	M	H	L	0	80%	20%	0	0
60	M	M	M	H	M	M	H	L	L	70%	20%	10%	0	0
61	M	L	M	L	H	H	M	M	H	20%	80%	0	0	0
62	L	M	H	M	M	H	L	L	L	30%	70%	0	0	0
63	H	H	M	M	H	L	M	M	H	0	40%	60%	0	0
64	M	H	M	L	H	H	H	VH	VH	0	0	70%	30%	0
65	L	H	H	VH	H	L	M	H	M	0	50%	30%	20%	0
66	M	H	H	M	L	H	M	M	VH	0	30%	70%	0	0
67	H	M	H	H	H	L	L	M	H	20%	80%	0	0	0
68	L	M	H	M	H	H	H	VH	L	0	50%	50%	0	0
69	L	M	L	L	L	H	H	M	H	0	0	60%	40%	0
70	L	M	L	L	M	L	L	L	M	40%	60%	0	0	0
71	H	M	M	L	L	H	M	L	L	10%	80%	10%	0	0
71	H	L	L	L	M	H	H	L	H	0	90%	10%	0	0
72	L	H	H	M	H	H	H	VH	VH	0	0	10%	30%	60%
72	L	H	M	M	H	L	M	H	M	0	0	40%	60%	0
73	M	M	H	H	L	L	M	M	H	0	60%	40%	0	0
74	H	L	L	H	L	L	L	L	L	80%	20%	0	0	0
75	H	L	L	L	L	L	L	M	L	80%	20%	0	0	0
76	H	M	L	L	H	H	H	H	H	10%	70%	20%	0	0
77	M	H	H	H	L	L	M	H	H	0	40%	60%	0	0
78	H	H	M	M	L	H	L	L	H	0	80%	20%	0	0
79	L	L	H	H	M	H	H	L	H	0	70%	30%	0	0
80	L	H	H	VH	L	H	L	VH	VH	0	0	0	80%	20%
81	H	M	H	L	H	H	M	M	M	10%	10%	80%	0	0
82	H	L	L	H	H	M	H	L	L	40%	40%	20%	0	0
83	H	L	M	L	L	H	L	L	L	60%	40%	0	0	0
84	H	H	H	H	L	L	H	M	L	0	40%	60%	0	0
85	L	M	M	H	L	H	L	L	L	20%	60%	20%	0	0
86	M	L	M	H	M	M	L	L	H	10%	70%	20%	0	0
87	L	H	H	M	H	H	H	VH	VH	0	0	80%	20%	0
88	H	M	H	L	L	H	L	L	L	0	80%	20%	0	0
89	L	M	L	L	H	H	M	L	L	10%	70%	20%	0	0
90	M	M	H	M	L	L	M	L	L	40%	60%	0	0	
91	L	L	M	L	L	H	L	H	H	0	20%	80%	0	0
92	H	L	L	L	H	L	L	L	H	20%	80%	0	0	0
92	H	H	L	L	H	H	H	VH	VH	0	0	80%	20%	0
93	L	VH	H	VH	H	L	L	H	H	0	0	60%	40%	0
94	H	L	M	M	H	L	H	M	M	0	50%	50%	0	0
95	L	M	H	VH	L	H	L	H	VH	0	40%	60%	0	0
96	M	M	M	M	M	H	M	M	L	0	70%	30%	0	0
97	H	M	M	L	H	L	L	M	M	10%	50%	40%	0	0
98	L	L	H	L	H	M	M	H	H	0	40%	60%	0	0
99	H	H	L	M	H	L	H	M	L	0	50%	50%	0	0
100	H	M	L	L	H	M	L	L	L	40%	60%	0	0	0
101	L	M	H	H	M	L	H	H	H	0	20%	80%	0	0
102	H	L	L	L	L	L	L	H	L	70%	30%	0	0	0
103	H	L	L	L	H	L	L	L	L	90%	10%	0	0	0
104	H	H	L	L	H	L	L	L	L	60%	30%	10%	0	0
105	M	H	L	L	L	H	L	L	L	50%	50%	0	0	0
106	L	M	L	H	H	M	H	L	M	30%	70%	0	0	0
107	M	M	M	H	H	L	L	H	M	0	60%	40%	0	0
108	M	M	H	H	H	H	L	H	H	0	30%	70%	0	0
109	H	L	H	H	VH	H	H	VH	VH	0	0	50%	50%	0
110	L	VH	H	VH	VH	H	H	H	H	0	0	90%	10%	0
111	H	M	H	L	H	H	H	M	H	0	10%	90%	0	0
112	L	H	H	M	M	L	L	VH	L	0	10%	80%	10%	0
113	H	L	L	M	H	M	H	H	H	0	20%	80%	0	0
114	L	H	H	L	H	M	M	H	L	10%	20%	70%	0	0
115	H	H	L	L	M	H	M	H	L	30%	70%	0	0	0
116	L	H	H	L	L	H	M	H	VH	0	60%	40%	0	0
117	M	H	M	M	VH	H	H	H	L	0	70%	30%	0	0
118	L	H	H	VH	H	L	M	H	VH	0	0	80%	20%	0
119	H	L	L	M	H	L	L	VH	L	0	80%	20%	0	0
120	L	H	H	M	M	H	L	L	L	20%	40%	40%	0	0
121	H	H	M	L	L	H	H	L	H	0	80%	20%	0	0
122	L	H	H	M	H	L	H	VH	M	0	40%	60%	0	0
123	H	M	L	L	H	L	L	L	L	20%	80%	0	0	0
124	M	M	M	H	H	M	L	L	L	10%	60%	30%	0	0
125	L	H	H	L	H	L	H	VH	H	0	0	90%	10%	0
126	H	L	H	M	H	L	L	VH	H	0	10%	80%	10%	0
127	M	M	M	M	M	M	M	L	L	10%	80%	10%	0	0
128	H	M	L	L	H	L	M	L	L	10%	90%	0	0	0
129	M	M	H	L	L	M	H	M	H	0	50%	50%	0	0
130	H	L	L	L	L	H	L	L	L	70%	30%	0	0	0
131	M	M	M	L	L	H	L	L	L	30%	40%	30%	0	0
132	H	M	M	L	H	L	H	L	L	10%	60%	30$	0	0
133	H	M	M	L	L	H	L	L	L	20%	80%	0	0	0
134	H	L	L	H	L	L	H	L	L	30%	70%	0	0	0
135	L	M	H	H	M	L	H	H	H	0	60%	40%	0	0
136	L	H	H	VH	VH	H	L	L	VH	0	20%	80%	0	0
137	H	L	L	H	M	H	H	VH	M	0	0	60%	40%	0
138	L	H	M	H	VH	H	M	M	H	0	20%	70%	10%	0
139	L	H	H	VH	M	H	M	L	VH	0	10%	90%	0	0
140	H	L	M	H	M	H	L	H	M	0	50%	50%	0	0
141	L	H	H	H	H	M	H	M	H	0	30%	70%	0	0
142	M	H	H	VH	H	L	H	H	VH	0	20%	80%	0	0
143	L	H	H	M	H	H	L	H	H	0	20%	50%	30%	0

## Abbreviations

*M*: Number of indicators
*L*: Number of belief rules
*N*: Number of consequents
$D_n$: Classification of the *n*th consequent
$\beta _{n,k}$: Belief degree associated to the *n*th consequent in *k*th belief rule
$\theta _k$: Weight of *k*th belief rule
$\omega _k$: Activation weight of *k*th belief rule
PMN: Primary membranous nephropathy
MN: Membranous nephropathy
NS: Nephrotic syndrome
IMN: Idiopathic membranous nephropathy
SMN: Secondary membranous nephropathy
ROC: Receiver operating characteristic
AUC: Area under curve
BRBS: Belief rule-based system
BRB: Belief rule base
FRBS: Fuzzy rule-based system
TFKNN: Tuned fuzzy kNN based on uncertainty classifiers
AI: Artificial intelligence
ALB: Albumin
TC: Total cholesterol
TG: Triglycerides
BUN: Blood urea nitrogen
CREA: Creatinine
eGFR: Estimated glomerular filtration rate
Urine SG: Urine specific gravity
Urine RBC: Urine red blood cells
GBM: Glomerular basement membrane
